# Development of an automated platform for the optimal production of glycoconjugate vaccines expressed in *Escherichia coli*

**DOI:** 10.1186/s12934-021-01588-1

**Published:** 2021-05-24

**Authors:** Jasmin J. Samaras, Marta Mauri, Emily J. Kay, Brendan W. Wren, Martina Micheletti

**Affiliations:** 1 Advanced Centre for Biochemical Engineering, University College London, Bernard Katz Building, Gower Street, London, WC1E 6BT UK; 2 Department of Infection Biology, London School of Hygiene and Tropical Medicine, Keppel Street, London, WC1E 7HT UK

**Keywords:** Automation, Screening studies, PGCT, Bioconjugation, Glycoconjugate vaccine, Pneumococcal vaccine, High throughput process development, Glycoprotein, Glycoengineering

## Abstract

Protein Glycan Coupling Technology (PGCT) uses purposely modified bacterial cells to produce recombinant glycoconjugate vaccines. This vaccine platform holds great potential in this context, namely due to its modular nature, the simplified production process in comparison to traditional chemical conjugation methods, and its amenability to scaled-up operations. As a result, a considerable reduction in production time and cost is expected, making PGCT-made vaccines a suitable vaccine technology for low-middle income countries, where vaccine coverage remains predominantly low and inconsistent. This work aims to develop an integrated whole-process automated platform for the screening of PGCT-made glycoconjugate vaccine candidates. The successful translation of a bench scale process for glycoconjugate production to a microscale automated setting was achieved. This was integrated with a numerical computational software that allowed hands-free operation and a platform adaptable to biological variation over the course of a production process. Platform robustness was proven with both technical and biological replicates and subsequently the platform was used to screen for the most favourable conditions for production of a pneumococcal serotype 4 vaccine candidate. This work establishes an effective automated platform that enabled the identification of the most suitable *E. coli* strain and genetic constructs to be used in ongoing early phase research and be further brought into preclinical trials.

**Supplementary Information:**

The online version contains supplementary material available at 10.1186/s12934-021-01588-1.

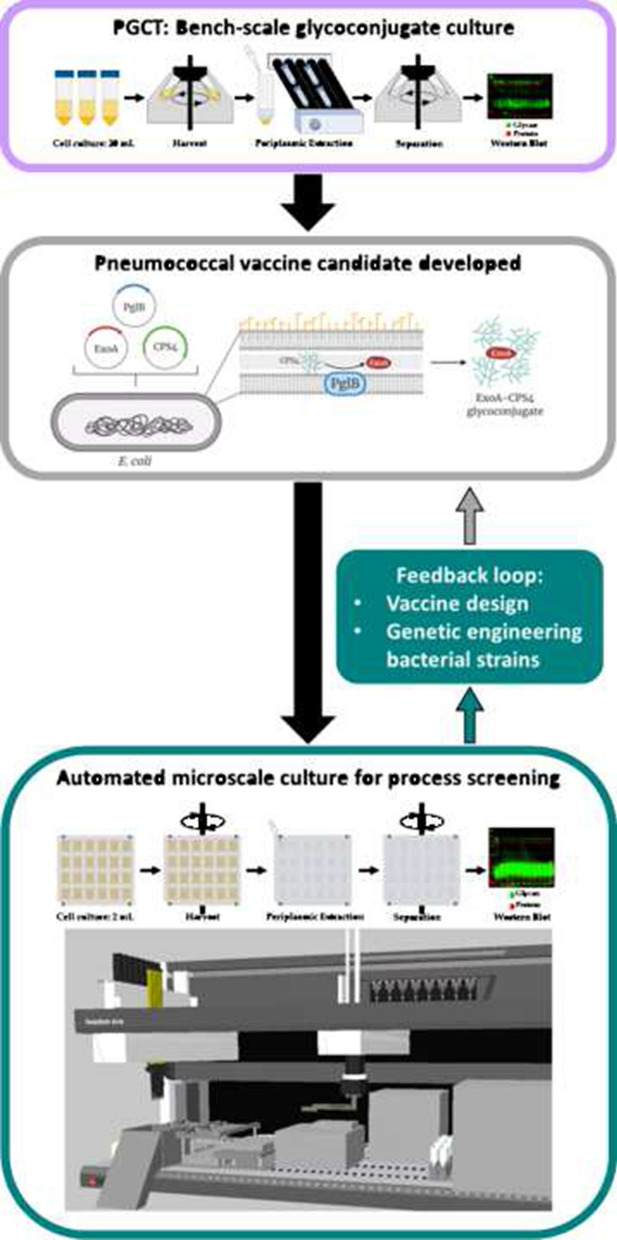

## Introduction

Vaccines are successful prophylactics that have greatly reduced the burden of disease worldwide. Conservative estimates from the World Health Organisation (WHO) suggest that vaccination saves at least 2–3 million lives every year [1], thus providing an invaluable cost-effective strategy in infectious disease prevention. Numerous vaccine technology platforms exist, these mainly include whole cell live attenuated vaccines (LAVs); inactivated vaccines; and subunit vaccines, where a few elements of the targeted pathogen trigger an immune response. Glycoconjugate vaccines are subunit vaccines consisting of a cell surface glycan, such as capsular polysaccharides (CPS), or O-antigens, covalently linked to an immunogenic carrier protein. These are amongst the safest and most efficacious vaccines in use today. In contrast to polysaccharide subunit vaccines, which are partially immunogenic in adults only, glycoconjugates effectively confer protection in both children and the elderly, two categories most ‘at-risk’ from infectious disease [2–7]. Pivotal research from Avery and Goebel in the 1930s [3, 4], together with the success of glycoconjugates introduced in the 1980s [5–7], has highlighted the importance of conjugation between the glycan and protein moiety in determining the immunogenicity of the vaccine.

The current production process of conjugate vaccines is a complex, multi-step process involving separate cultivations of a pathogenic strain to harvest glycan antigens, and of a second strain expressing the carrier protein. Two distinct purification chains are then needed before pre-treatment of the two components for chemical conjugation. This is followed by a final purification chain to obtain the purified glycoconjugate. The need for at least three full purification sequences makes production of glycoconjugate vaccines costly, time consuming, prone to product loss and often yields contaminated preparations and batch to batch variation, given the random nature of chemical coupling. Due to these inefficiencies, vaccine coverage in predominantly low-middle income countries (LMICs) remains inconsistent [1]. For example, in 2017 pneumonia was responsible for the deaths of 2.56 million people, a third of whom were below 5 years, despite there being effective vaccines on the market targeted against *Streptococcus pneumoniae,* the leading cause of pneumonia [8–10]. Pneumococcal conjugate vaccines are unaffordable for many LMICs that lose support of the Global Alliance for Vaccines and Immunisation (GAVI) for reduced pricing ($3.05–$169 /dose for GAVI-supported countries to high income countries) [11]. Moreover, the geographic distribution of pneumococcal serotypes varies and serotypes not covered by the current vaccines are increasingly causing disease, a phenomenon known as serotype replacement [12, 13]. This further increases the demand for affordable vaccines that can be easily tailored to the targeted area/population.

Bacterial genetic engineering has allowed the recent development of Protein Glycan Coupling Technology (PGCT), which relies on the in vivo coupling of recombinant glycan antigens to an immunogenic carrier protein when co-expressed with an oligosaccharyltransferase (OST) enzyme in an *E. coli* cell (reviewed in [14, 15]). With an inexhaustible and homogenous preparation of the glycoconjugates formed within the purposely engineered strains of *E. coli*, the overall production process is simplified to a single purification sequence from cell harvest. This is expected to considerably reduce production costs, thus making PGCT a suitable vaccine technology for LMICs and for the veterinary market. PGCT-made glycoconjugates have shown success both in preclinical and clinical trials, for an exhaustive overview see Table 3 in [14], however no streamlined production platform is publicly available. One of the biggest hurdles to vaccine commercialisation is the high demand on time and resources. During vaccine development, the research and development phase alone is estimated to last up to 5 years and include hundreds of candidates. The duration of this phase can vary greatly and incorporates the establishment of a vaccine antigen/identity and the production process [17]. During an epidemic or pandemic scenario rapid response in finding the right vaccine candidate is vital and timescales must be vastly reduced, as seen with the recent SARS-CoV-2 outbreak. PGCT holds great potential in this context given its modular nature, the simplified production process and its amenability to scaled-up operations. In addition, automated screening methodologies have proven invaluable for improved productivity and reproducibility [18–20], however a balance is often needed between flexibility and complexity to attain a precision platform that can suitably adapt to the inherent biological variation associated with cell culture.

The rapid and reliable screening of novel vaccine candidates is central to minimising costs and to speed up the research and development phase in vaccine manufacture. The process developed is equally critical as the product, and both must be optimised for successful manufacturing. Recognising this at an international level, the IPROVE (Innovation Partnership for a Roadmap on Vaccines in Europe) FP7 project drew attention to the potential benefits of building a fully multi-disciplinary vaccine research community. The FP7 project, covering vaccine discovery, development, production and access, set out 80 key recommendations to achieve its mandate of affordable, faster, more flexible and less wasteful vaccine production in Europe. Among the recommendations, there was a recurring theme of close collaboration needed amongst scientists, engineers and regulators for improvement in vaccine manufacturing, innovation and quality control [16]. Importantly, this work represents a close collaboration between molecular biologists and biochemical engineers for vaccine design and process development. We present an integrated whole-process automated platform for the screening of PGCT-made glycoconjugate vaccine candidates, aimed for eventual transfer to a low-income setting. The platform will be accessible to non-automation experts to deploy an automated workflow and will provide a powerful tool in the early research and development phase of this work.

## Materials and methods

### Bacterial strains, growth conditions and plasmids

Electrocompetent *Escherichia coli* K-12 strains (W3110, CLM37, CLM24, SDB1 and Falcon) were transformed with plasmids encoding the three PGCT components (glycan, carrier protein and PglB OST) in 1 mm gap cuvettes at 2 kV, 200 Ω and 25 µF. Strains were grown in Lysogeny Broth (LB) or on LB agar at 37 °C with the required antibiotics at the following concentrations: 100 µg/mL ampicillin, 20 µg/mL tetracycline and 50 µg/mL kanamycin. *E. coli* DH5α were used as a host for cloning experiments. Colony forming units (CFUs) of PglB-induced and uninduced Falcon cultures were determined in biological triplicates. Briefly, cultures were matched at an optical density measured at 600 nm (OD_600_) and tenfold serial dilutions were performed for each sample (10^0^–10^–7^). 2 µL of each dilution were spotted in technical triplicates on dry LB agar plates containing the appropriate antibiotics. Colonies were counted after O/N incubation at 37 °C and CFU/mL were calculated.

*Pseudomonas aeruginosa* exotoxin A was modified according to [21] to obtain a detoxified version containing two internal PglB glycosylation sequons. The genetic sequence encoding for the modified protein was codon optimised for *E. coli* and ordered via gene synthesis from GenScript. The sequence was N-terminally fused to either DsbA or PelB signal peptide for secretion to periplasm, and a 6xHis tag was added C-terminally. DsbA-ExoA(10) has eight extra optimal PglB glycosylation sequons (DQNAT), four added N-terminally downstream of the signal peptide and four added C-terminally upstream of the His-tag spaced by “GG” duplets. The sequence was also ordered via gene synthesis from GenScript. Constructs were subcloned into pEC415 or pEXT20 vector via restriction enzyme digest using NheI-EcoRI and EcoRI-BamHI, respectively (High Fidelity versions, New England Biolabs). Details on all strains and plasmids used in this study can be found in Additional file 1: Tables S1 and S2, respectively.

All strains used during the strain screening in ‘Application of the automated platform for screening studies' section are referred to as in Table 1.

**Table 1:** Identifiers used for the various strains of *E. coli* studied

Strain	W3110	CLM37	CLM24	SDB1	Falcon	
Plasmids	+ pB4 + pEXT22-PglB + pEC415-ExoA(10)				+ pB4 + pEXT22-Ø + pEC415-ExoA(10)	+ pB4 + pEXT22-PglB + pEC415-ExoA(10)
Identifier	Strain 1	Strain 2	Strain 3	Strain 4	Strain 5	Strain 6

### Inoculum preparation

A 1 mL 40% glycerol working cell bank was used to inoculate a 50 mL falcon tube containing 10 mL of LB, supplemented with the antibiotics listed in “Bacterial strains, growth conditions and plasmids” section. The preculture was incubated at 37 °C overnight (O/N, ≈ 16 h) with agitational speed (*N* = 180 rpm) on an orbital shaker platform (orbital diameter, *d*_*o*_ = 25 mm). Unless otherwise specified, all reagents used in this work were obtained from Sigma-Aldrich, Switzerland.

### Production of glycoconjugates at the bench scale

**Fig. 1:**
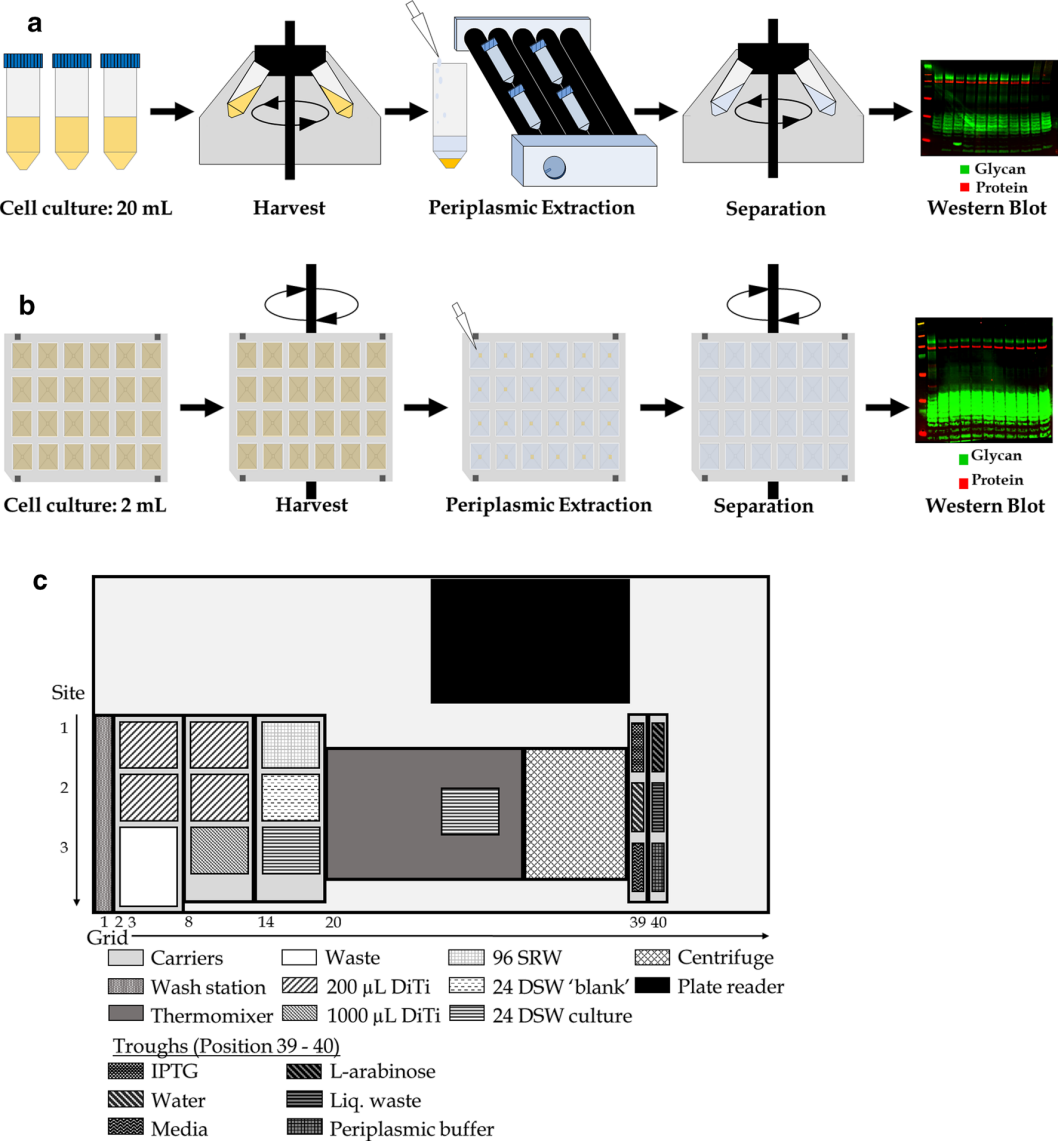
**a**-**b** Process steps for glycoconjugate production at the bench scale (**a**) and the microscale (**b)**. **c** Top-view schematic representation of the automated worktable depicting the carriers and labware, including disposable tips (DiTis), 96-standard round well plate (SRW) and 24-deep square well plate (DSW)

For production of glycoconjugate at the bench scale, cell culture was performed in cylindrical falcon tubes with a conical bottom geometry. The preculture was used to inoculate 20 mL of LB media, supplemented with the antibiotics listed in “Bacterial strains, growth conditions and plasmids” section, to a liquid height, *H*_*L*_ = 45.5 mm and an OD_600_ of 0.045. Cultures were grown at 37 °C, *N* = 180 rpm to an OD_600_ of 0.6–0.8 (late exponential phase). Expression of PglB and ExoA were induced with the addition of 1 mM IPTG and 0.4% (w/v) l-arabinose, respectively, before the culture was grown at 28 °C for a further 20 h on an orbital shaker platform. Cells were then harvested by centrifugation for 15 min at 3000×*g* at either 4 °C or 22 °C, depending on which subsequent periplasmic buffer was used (lysozyme-based method or heat-based method), as described in “Periplasmic extraction” section. The supernatant was discarded, and the cell pellets were resuspended in periplasmic buffer, according to the protocols detailed in “Periplasmic extraction” section. Following periplasmic extraction, a final centrifugation step was performed at 5000×*g* and 4 °C for 15 min and the supernatant used for western blot analysis. Figure 1a illustrates the bench scale production platform.

### Production of glycoconjugates at the microscale

For production of glycoconjugate at the microscale, cell culture was performed in 24-deep square well plates (24-DSW). Each well has a square geometry and a truncated pyramid bottom. The preculture was used to inoculate 2 mL/well of LB media, supplemented with the antibiotics listed in “Bacterial strains, growth conditions and plasmids” section to a liquid height, *H*_*L*_ = 6.9 mm and an OD_600_ of 0.045. To limit liquid evaporation and cross contamination between the wells, whilst ensuring sufficient oxygen transfer, Duetz-system sandwich covers were used (Kuhner Shaker, Switzerland). The culture was grown at 37 °C on a Thermomixer Comfort agitated at *N* = 300 rpm (*d*_*o*_ = 3 mm) to an OD_600_ of 0.6–0.8 (Eppendorf, Germany). Expression of PglB and ExoA were induced with the addition of 1 mM IPTG and 0.4% (w/v) l-arabinose, respectively, before the culture was grown for 20 h at 28 °C, *N* = 300 rpm. Each plate was then centrifuged for 15 min at 4300 × g and at 4 °C or 22 °C, depending on which subsequent periplasmic buffer was used, as described in “Periplasmic extraction” section. The supernatant was removed, and the remaining cell pellets were resuspended in periplasmic buffer, according to the protocols detailed in “Periplasmic extraction” section. For Periplasmic Buffer 1 (PB1) it was necessary to transfer the resuspended cell pellets from each well to separate tubes for agitation on a roller mixer at 4 °C. Periplasmic Buffer 2 was used to resuspend the cell pellets in situ and agitation/heating was achieved using the thermomixer. Following periplasmic extraction, a final centrifugation step was performed at 5000×*g* and 4 °C for 15 min and the supernatant used for western blot analysis. Figure 1b illustrates the microscale production platform.

### Periplasmic extraction

Following cell harvest, the resultant pellet from each culture condition was resuspended in periplasmic extraction buffer to a matched OD_600_ value of 20 per tube or well. This ensured that cell biomass prior to periplasmic extraction was normalised across all samples. Periplasmic extracts were prepared using either lysozyme treatment, as described in Feldman et al***.*** (Periplasmic buffer 1, PB1), or a heat-based method from Bowering et al*.* (Periplasmic buffer 2, PB2) [22, 23]*.* The lysis buffer (PB1) was composed of an initial preparation of 30 mM Tris–HCl pH 8.5, 1 mM EDTA and 20% (w/v) sucrose. This was then sterile filtered (0.22 µm, UltraCruz® Syringe Filter) and fresh additions of lysozyme (1 mg/mL) and cOmplete™ mini EDTA-free protease inhibitor cocktail (1 tablet/10 mL, Roche, Switzerland) were made before incubation at 4 °C for 0.5 h with agitation. Periplasmic extraction buffer 2 (PB2) was composed of sterile filtered 100 mM Tris–HCl pH 7.4 and 10 mM EDTA and samples were incubated for 1 h with agitation at 30 °C. The composition of each periplasmic buffer is summarised in Table 2. To improve upon periplasmic extraction yields, a range of alternative combinations of temperature (*T*) and incubation time (*t*) were investigated, these are given in Table 3.

**Table 2:** Composition of Periplasmic Buffers (1) and (2)

Components	Periplasmic Buffer 1	Periplasmic Buffer 2
Tris.HCl	30 mM (pH 8.5)	100 mM (pH 7.4)
Sucrose	20% (w/v)	-
EDTA	1 mM	10 mM
Lysozyme	1 mg/mL (fresh)	-
Protease inhibitor cocktail	1 tablet/10 mL (fresh)	-

**Table 3:** Periplasmic extraction methods tested

Condition^a^	*t* [h]	*T* [°C]	Protease inhibitor [Y/N]
Control	0.5	4	Y
1A	0.5	4	N
1B	1	4	Y
1C	1	4	N
1D	0.5	22	Y
2A	O/N	60	Y
2B	O/N	22	Y
2C	O/N	22	N
2D	O/N	30	Y
2E	O/N	30	N
2F	1	60	Y
2G	1	60	N
2H	1	45	Y
2I	1	45	N
2 J	1	30	Y
2 K	1	30	N
2L	1	22	Y
2 M	1	22	N

^a^The prefix for each condition represents Periplasmic Buffer 1 or 2. The subsequent letter (A–M) distinguishes the different conditions, changing either duration (*t*), temperature (*T*) or protease inhibitor tablet addition during periplasmic extraction. ‘O/N’ shows overnight incubation of ~ 16 h

### Fluorescent western blotting

To verify glycoconjugate production, periplasmic extracts were analysed by SDS-PAGE and western blotting. Equal volumes of samples, previously normalised by optical density, were used. This enabled semi-quantitative comparison between samples on one blot. Samples were initially prepared with LDS sample buffer and separated on 4–12% bis–tris gels in MOPS buffer (Invitrogen, USA). The gels were then electroblotted onto a nitrocellulose membrane using a Trans-Blot Turbo Transfer System (Bio-Rad, USA). Rabbit anti-serotype 4 capsule antibody (Statens Serum Institut, Denmark) was used at a dilution of 1:1000 and mouse anti-His monoclonal antibody (Thermo Fisher Scientific, USA) was used at a dilution of 1:10,000 to detect recombinant serotype 4 capsule and His-tagged ExoA, respectively. Secondary goat anti-rabbit IgG IRDye 800 and goat anti-mouse IgG IRDye 680 conjugates (LI-COR Biosciences, USA) were used at a dilution of 1:10,000. Fluorescent signal in two channels, 700 and 800 nm, was detected with an Odyssey CLx LI-COR detection system, using a solid-state diode laser at wavelengths 685 and 785 nm, respectively (LI-COR Biosciences, USA). Subsequent semi-quantitative densitometry analysis of the glycoconjugates was performed using the Image Studio (LI-COR Biosciences, USA) analysis tool, as demonstrated in the supplementary material (Additional file 2: Figure S1).

### Sandwich ELISA

Semi-quantitative analysis of ExoA-CPS4 glycoproteins in periplasmic extracts matched by optical density were performed by sandwich ELISAs with a protocol modified from [24]. Transparent polystyrol 96-well plates with high protein binding capacity (F96 MaxiSorp, Nunc) were coated with goat polyclonal *P. aeruginosa* Exotoxin A antibody (Fitzgerald, USA) diluted 1:1000 in PBS (100 µL/well) and incubated overnight at 4 °C. Wells were then washed four times with 200 μL PBS-T (0.1% Tween-20) for 2 min static and 2 min 500 rpm, and blocked for at least 2 h at room temperature with 200 μL 10% (w/v) milk in PBS-T per well. After blocking, wells were washed twice as described above and 60 µL/well of periplasmic extract was added to the plate and incubated overnight at 4 °C. Prior to addition to the plate, periplasmic extracts were diluted 1:100 in 50 mM Na_2_CO_3_ pH 9.6 and treated with 2% Triton X-114 to remove lipid-bound CPS4 as described in [25]. Unbound ExoA was removed with four washes performed as described above. To quantify the CPS4 glycan and ExoA protein content of ExoA-CPS4 glycoproteins rabbit anti-CPS4 antiserum (Statens Serum Institut, Denmark) and mouse monoclonal anti-His (Thermo Fisher Scientific, USA) were added to separate wells of duplicated samples at a 1:1000 dilution in 1% (w/v) milk in PBS-T. The plate was then incubated for 2 h at room temperature, *N* = 500 rpm. Unbound antibodies were removed by four washes as previously described. Detection antibodies goat-anti-rabbit IgG-HRP (Abcam, UK) and rabbit-anti-mouse IgG-HRP (Sigma-Aldrich, Switzerland) were added at 1:10,000 dilution in 1% (w/v) milk in PBS-T, incubated for 1 h at room temperature, and unbound antibodies were removed by four washes. ELISA plates were developed with 100 µL/well TMB substrate solution (Invitrogen, USA). The oxidative reaction was stopped by adding 100 µL/well H_2_SO_4_ (2 N) and absorbances at 450 nm were detected using a SpectraMax iD5 plate reader (Molecular Devices, USA). OD_450_ background values (buffer only in wells treated as the rest, or proteinase K-treated extracts) were subtracted from test values, triplicates or hexaplets were averaged and error bars represent standard deviations. All ELISAs were performed in duplicate.

### Proteinase K digestion

50 µg/mL of proteinase K (10 mg/mL) was added to periplasmic extracts to digest their proteome. Samples were incubated in a water bath at 55 °C for 1 h, after which they were used for downstream analysis.

### Automated microscale platform

A Tecan Freedom Evo 150 base station programmed and controlled by Freedom EVOware® version 2.6 was equipped with an eight-channel liquid handling (LiHa) arm and an eccentric robot manipulator (RoMa) arm (Tecan, Switzerland). The LiHa was fitted with disposable tips (DiTi) and the RoMa was used for plate manipulation around the platform. Figure 1b depicts the sequence of operations to be performed on the platform, excluding the western blot analysis. The platform was integrated with an Infinite M200 plate reader, operated by Magellan™ software version 7.2 (Tecan Group Ltd.), a Rotanta 46 RSC microwell centrifuge (Hettich, Germany) and a Thermomixer Comfort (Eppendorf, Germany), all of which were enclosed within a Class II biosafety cabinet.

A top-view representation of the worktable is given in Fig. 1c, showing the positions of all the carriers and labware required for a complete production run. It is worth noting that in total two 24-DSW plates were used, one for a balance plate during centrifugation and one used for culture, which was moved by the RoMa between the thermomixer for heated agitation and the carrier for larger volume additions (> 200 µL), corresponding to grid positions 20 and 14, site 3, respectively (see Fig. 1c).

An EVOware script was developed for ‘walk-away’ unsupervised operation for all process steps, excluding western blotting (Fig. 1b). The script includes conventional commands and worklists, and is able to self-execute applications for calculation, decision-making, worklist creation and report generation of biomass growth over the course of cell culture. The executable application was created using a purposely written Matlab code and compiled using the Matlab Compiler™ (Mathworks, USA, release 2019b).

## Results and discussion

### Identification of a scalable periplasmic extraction methodology for PGCT-made glycoconjugate vaccines

The aim of this work was the development of an automated microscale screening platform for PGCT-made glycoconjugate vaccines, currently obtained from a bench scale production process. The prototype glycoconjugate vaccine analysed in this study is a pneumococcal vaccine against serotype 4 composed of carrier protein ExoA covalently attached to the capsular glycan of serotype 4 pneumococcus. More specifically, a genetically detoxified variant of *P. aeruginosa* Exotoxin A N-terminally fused to a signal peptide for periplasmic expression, C-terminally fused to a hexahistidine tag and genetically engineered with *N*-glycosylation sites was used [26, 27]. The presence of glycosylation sequons turns the modified ExoA into a substrate for *N*-linked glycosylation with *S. pneumoniae* capsular polysaccharide of serotype 4 (CPS4) when co-expressed with the oligosaccharyltransferase PglB from *Campylobacter jejuni*, which functions in the periplasm of gram-negative bacteria. Previous work has demonstrated CPS4 bioconjugates to confer protection against a homologous pneumococcal strain in murine models, and the carrier protein ExoA to be a suitable candidate for production at scale [26, 28, 29].

**Fig. 2:**
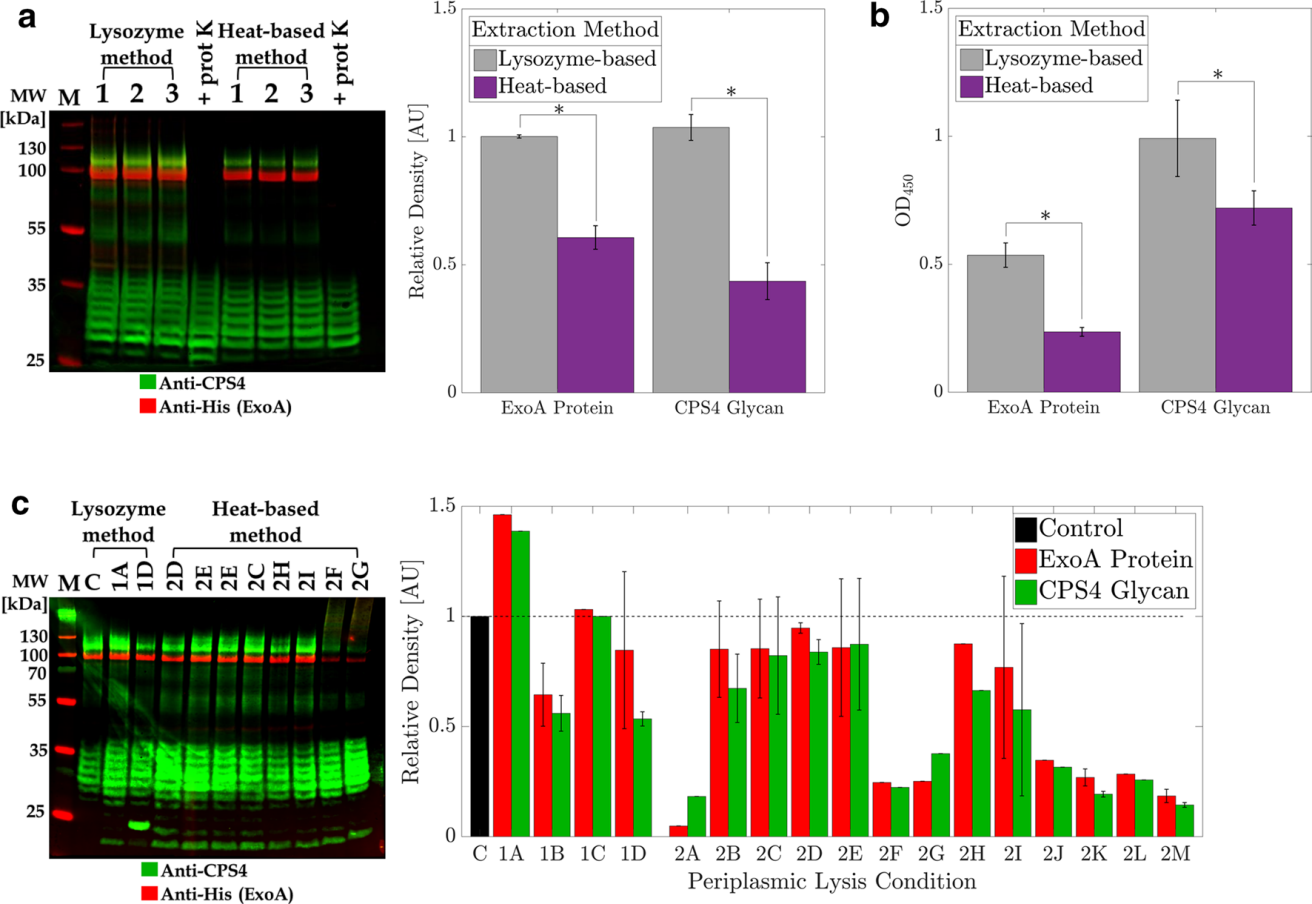
Comparison of different periplasmic extraction methods. **a** Western blotting and densitometry analysis showing the presence and recovery of ExoA-CPS4 glycoproteins obtained with both the lysozyme and heat-based methods of periplasmic extraction. Lanes 5 and 9: Proteinase K digests. Relative quantification based on pixel intensity; **b** Semi-quantitative sandwich ELISA analysis of ExoA-CPS4 glycoproteins in periplasmic extracts. ExoA-coupled CPS4 glycan and ExoA protein (His) were quantified independently. Statistical significance (*) using an unequal variance two-tailed t-test is considered in all experiments when *p* ≤ 0.05; **c** Western blotting showing the presence of ExoA-CPS4 glycoproteins in periplasmic extracts obtained with some of the conditions listed in Table 3 and densitometry results of protein (red) and glycan (green) quantities based on pixel intensity normalised to the control condition

To develop the screening platform, all process steps needed to first be translated to the microscale and be amenable to automation. The main bottleneck was the lysozyme-based periplasmic extraction step, which although could be scaled down, could not be performed without manual input, namely due to the fresh additions of lysozyme and protease inhibitor enzymes. Additionally, the lysozyme-based extraction protocol required a cold incubation step at 4 °C, which was problematic on the automated platform. Both the fresh additions of enzymes and the requirement for cold incubation are better avoided when considering eventual scale-up of the process due mostly to their high cost. A number of alternative methodologies were found in the literature and a simple heat-based methodology, introduced in the work of Bowering et al*.* [23], was considered the most appropriate. The protocol, outlined in “Periplasmic extraction” section, was amenable to both automation and large scale processes and had been proven to work for extraction of antigen-binding fragments (Fab’) at a range of temperatures [23, 30]. An initial investigation between the ‘control’ condition, consisting of a sample that underwent lysozyme-based extraction for 0.5 h incubated at 4 °C with a protease inhibitor tablet added (see Table 3), and a sample that underwent heat-based extraction, incubated for 1 h at 30 °C (condition 2 K, Table 3), is presented in Fig. 2a and b. Each condition was performed in triplicate before the periplasmic extracts were separated by SDS-PAGE and analysed by western blotting, shown in Fig. 2a. Bearing in mind the later application of the protocol to microscale cultures, a heavily glycosylatable version of ExoA with 10 glycosylation sequons was used to maximise glycosylation detection (similar to Marshall et al*.* [27]). ExoA(10)-CPS4 glycoproteins run at ≈ 100 kDa (lanes 2–4 and 6–8 of Fig. 2a). Lanes 5 and 9 show the sample following digestion with proteinase K, where the absence of the glycoconjugate confirmed the anchorage of the CPS4 glycan to the ExoA carrier protein. The CPS4 visible at the bottom of the blot is still detected in proteinase K-treated samples indicating it is not protein-bound. An initial comparison between the two periplasmic extraction techniques, based upon semi-quantitative densitometry analysis/pixel intensities from the western blotting, showed a statistically significant 40–60% reduction in product yield using the heat-based extraction protocol (Fig. 2a, right). This reduction in product yield was confirmed using a second semi-quantitative technique, a sandwich ELISA measuring ExoA-CPS4 glycoprotein from periplasmic extracts. The ELISA showed a 30% decrease in CPS4 glycan coupled to ExoA and ≈ 56% decrease in the extracted ExoA protein with the heat-based extraction protocol in comparison to the lysozyme-based extraction method (Fig. 2b). The discrepancies observed between the densitometry and ELISA estimates are as a result of the advantages and limitations associated with each method. ELISA assays are highly sensitive and specific, with a readout based on a time sensitive enzyme/substrate reaction. Densitometry analysis of fluorescent blots are time-independent, however are less sensitive to weaker signals when masked by stronger intensities. Both methods were adopted to independently validate our observations. Importantly, despite the difference in estimates, conclusions drawn from both methods are in agreement. Whilst product recovery was reduced, this revealed a potential method for simplified periplasmic extraction, amenable to both automation and scale-up. The glycan to protein ratio in the recovered product was also increased. To investigate whether improved recovery could be achieved, a range of conditions were tested, summarised in Table 3. Incubation temperature was varied between 22 °C (room temperature)–60 °C with either overnight (≈ 16 h) or 1 h incubation periods. Figure 2c shows a western blotting comparing multiple periplasmic extraction conditions. Through densitometry analysis it was possible to estimate the variation in periplasmic extraction performance for all the conditions tested (see Table 3). Figure 2e gives the relative yield of the conditions investigated, where the prefix for all conditions represents either Periplasmic Buffer 1 (lysozyme-based method) or Periplasmic Buffer 2 (heat-based method). Considering the best performing condition for the lysozyme-based extraction protocols (condition 1A), an approximate 40% increase in product recovery was achieved in comparison to the control. In both cases the sample was incubated at 4 °C for 0.5 h with and without the addition of the protease inhibitor cocktail (control and sample 1A, respectively). Interestingly, the addition of the protease inhibitor cocktail reduced variability when used with the heat-based periplasmic extraction methodology, whilst product expression remained mostly unchanged. The fresh addition could not be achieved on an automated platform however, and it is generally preferable at larger scales to exclude such costly additions, thus conditions without protease inhibitors were selected. Of the heat-based extraction conditions investigated (Fig. 2c), three conditions were shown to provide relatively good product recovery in comparison to the control. For overnight incubation (≈ 16 h) and heating to either 22 °C (2C) or 30 °C (2E) product recovery reached approximately 82% or 87% of the control condition, respectively. Shorter incubation times for 1 h at 45 °C (2I), whilst not as effective, still achieved approximately 60% product recovery in comparison to the control. Overall, although a reduction in product recovery was observed, a viable alternative methodology was found which was simpler to perform, amenable to automation and feasible to use with process scale-up.

### Establishment of an automated screening platform

#### Manual process translation and automated platform development

**Fig. 3:**
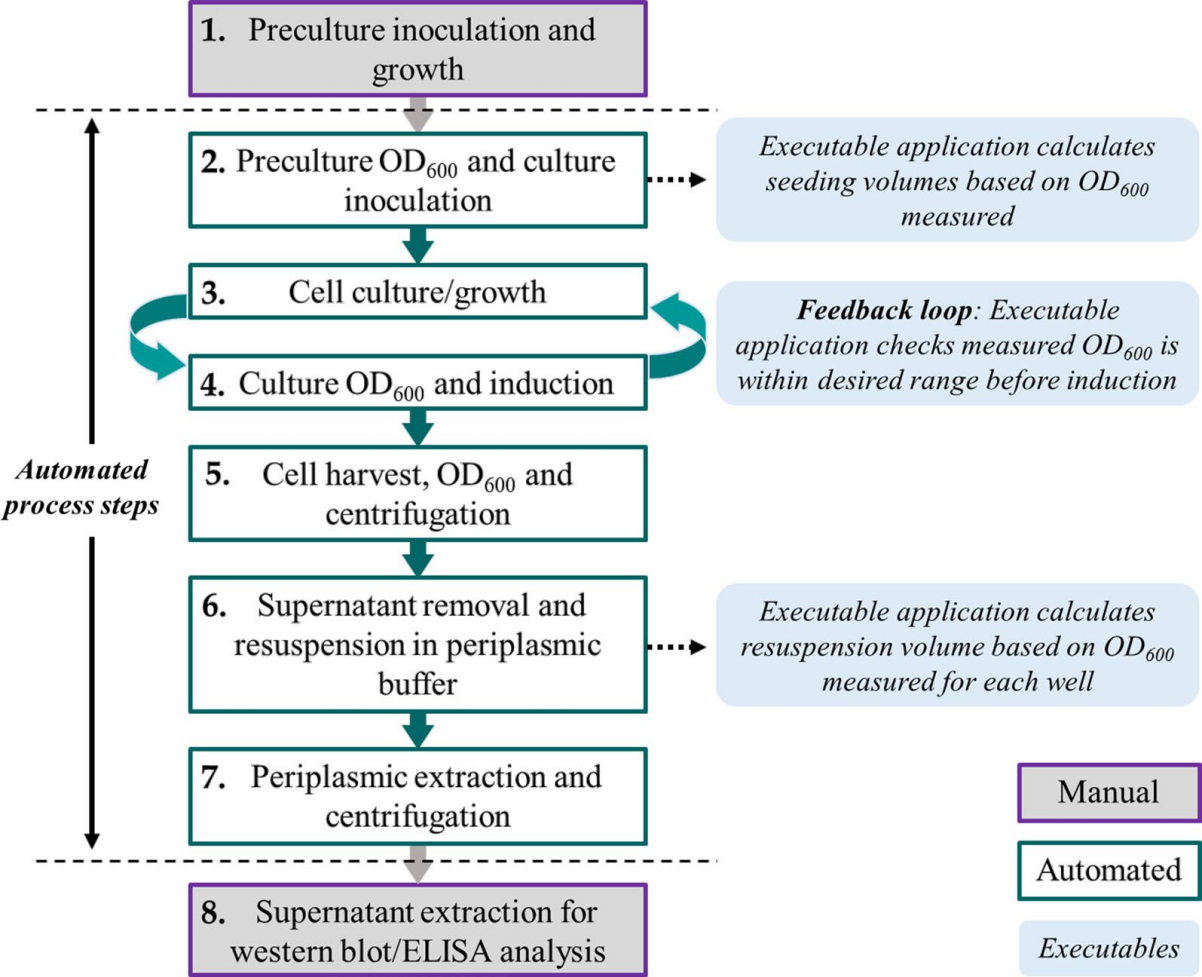
Process flow of the operations performed on the automated platform. Executable applications and feedback loops are indicated in *italics*

At this point, a small-scale equivalent had been established for each of the five process steps of glycoconjugate production (Fig. 1b). In order to create an automated sequence of operations for the platform, the microscale process, which was split into five discrete steps, had to be linked and scripts for the various stages of glycoconjugate production were defined. The first production stage depicted in Fig. 1b, ‘Cell culture’, was considered as four distinct sub-sections, as shown in Fig. 3. These sub-sections separated the initial manual handling and setup stage of the preculture (Step 1), where additional subsequent breakdown was needed to distinguish culture inoculation, cell growth and culture induction. This was mostly due to an integrated feedback loop within the script. Following Step 1, the operator loaded the preculture and all process solutions onto the automated worktable, as shown in Fig. 1c. During Step 2, the optical density measured at 600 nm (OD_600_) of a well-mixed preculture sample was measured, before launching an executable application which calculated the inoculation volume for the desired seeding concentration based upon the preculture OD_600_ value. The application exported the calculated inoculation volumes to a spreadsheet, which was then imported into EVOware and used for defining culture inoculation. The culture was then grown on the integrated thermomixer for a predefined duration (Step 3). A feedback loop was integrated between Steps 3 and 4 (Fig. 3), where the executable application was again launched following an OD_600_ measurement of the culture. The application performed a logical test to confirm if a given condition was satisfied, returning either a TRUE (1) or FALSE (0) value. In this case, the logical test checked whether a specified threshold in cell growth had been attained before culture induction. The result of the logical test was then imported into EVOware where two branches of action were possible. The value from the executable application (0 OR 1) determined whether the EVOware script either induced the culture (1, Step 4) or repeated cell growth (0, Step 3) for a shorter interval before another OD_600_ check was made. This loop was repeated hourly until the desired threshold in cell growth was achieved. Following induction and incubation (Step 4), the second production stage in Fig. 1b, ‘Harvest’, was scripted onto the automated platform. The harvest OD_600_ was measured for each well before the robotic manipulator arm transferred the plate to the integrated centrifuge (Step 5, Fig. 3). Once each well was pelleted down, the liquid handling arm removed the supernatant from each well and the third production stage (Fig. 1b), ‘Periplasmic extraction’, was performed by the robot (Step 6, Fig. 3). Following supernatant removal, EVOware launched the executable application, which calculated the resuspension volume in each well of the periplasmic extraction buffer to achieve a matched cell density. The application generated a worklist with multiple liquid handling commands, defining the source labware, the volume required to aspirate/dispense, the liquid class used, the destination labware and the well locations in the source and destination labware. This worklist was imported and then executed in EVOware before the wells were incubated on the thermomixer. After the specified incubation for periplasmic extraction, the script for the ‘Separation’ stage (Fig. 1b) was written. The microwell plate was transferred to the centrifuge where the resultant supernatant was kept, containing the product (Step 7, Fig. 3). The periplasmic supernatants were then analysed using SDS-PAGE, western blotting and ELISA (Step 8, Fig. 3), according to “Fluorescent western blotting” and “Sandwich ELISA” sections and the final production stage in Fig. 1b. From the automated workflow given in Fig. 3, Steps 2–7 are automated process steps, performed unsupervised by the robot over the course of three days. An operator loads the platform on Day 1, defining the variables for culture inoculation and induction, before returning on Day 3 to run SDS-PAGE/western blot analysis on the samples. The executable application also generated cell growth reports each instance it was launched over the three days.

The automated process was designed to exclude the need for any manual intervention during Steps 2–7, thus improving research output and efficiency in comparison to manual operations. The feedback loop and executable functions included in the script enable calculation and adjustment between culture runs to account for biological variability and ensure consistency across experiments. This is discussed in the following section. Once established, the platform can be used by non-automation experts and provides a powerful tool for parallel hands-free screening of different conditions.

#### Evaluation of the automated platform robustness

**Fig. 4:**
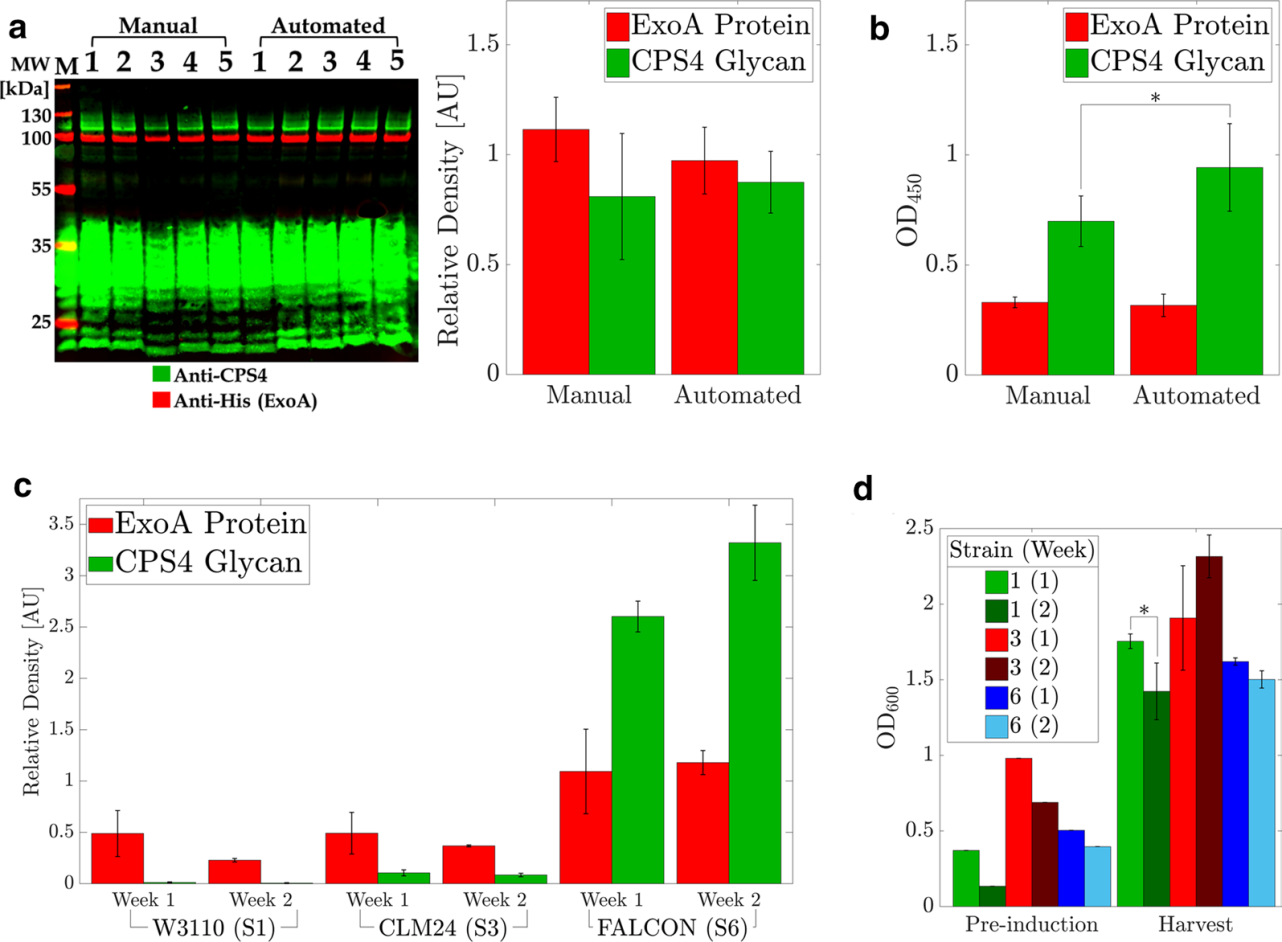
Platform robustness and comparability. Technical repeatability investigation between the manual and automated handling platforms; **a** Western blotting and subsequent densitometry analysis showing the presence of ExoA-CPS4 glycoproteins in periplasmic extracts; **b** Sandwich ELISA analysis of periplasmic extracts obtained from both manual and automated cultures; Biological repeatability investigation of three glycoconjugate-producing strains repeated over two separate experimental weeks; **c** Relative quantification of CPS4 glycan and ExoA protein based on pixel intensity normalised to a common control condition; **d** OD_600_ of cultures measured pre-induction and at harvest. Statistical significance (*) using an unequal variance two-tailed t-test is considered when *p* ≤ 0.05

Following the development of the automated platform this work then sought to prove technical repeatability and comparability of the automated and manual handling platforms. A proof-of-concept parallel comparison was made on a single 24-DSW where the same initial cell stock and thermomixer were used to minimise biological variation and variation in heat distribution with a different mixer. A total of 12 repeats per condition were completed, where the automated platform was paused to execute manual operations. Figure 4 shows the results of this investigation. An example western blotting of this comparison is given in Fig. 4a which shows the successful production of the bioconjugated ExoA-CPS4 in both platforms. Densitometry analysis of the ExoA protein and CPS4 glycan bands at ≈ 100 kDa, normalised to a control condition, showed similar performance between the automated and manual platforms in terms of final average product yield and harvested biomass (+ 26% achieved with manual handling). An ELISA analysis showed similar trends to the western blotting, giving approximately 35% higher glycan content and similar protein levels between the automated and manual handling platforms, thus resulting in an improved glycan to protein ratio in the product obtained from automated samples (Fig. 4b). The slight reduction in yield from the automated platform was likely due to biomass loss during the supernatant removal at the point of harvest, however a good level of consistency across the 12 repeats was achieved (15–28% standard deviation in product expression in comparison to 8–16% standard deviation from manual handling). This demonstrated the successful parallel production of glycoconjugate on the developed automated platform in addition to similar overall productivity to the manual operation achieved with minimal user input.

With any biological process there is an expected level of intrinsic variation. In repeating the automated screening across several weeks, it was possible to investigate the extent of biological repeatability on the platform, i.e. was similar performance achieved between different weeks when handled in the same manner. Three strains were investigated, all modified to express the glycoprotein of interest as detailed in “Bacterial strains, growth conditions and plasmids” section. The relative density of glycoconjugate expression from western blotting analysis and the harvested biomass yields are given in Fig. 4c, d, respectively. Similar performances between separate weeks are immediately apparent and were confirmed using a two-sample t-test (α = 0.05) between week 1 and week 2. No significant statistical difference was found for product expression (ExoA-CPS4, Fig. 4c) for any of the three strains considered. Similarly, no significant difference in OD_600_ values measured at induction and at point of harvest were detected between week 1 and week 2, with the exception of W3110 strain (strain 1) which exhibited ≈ 24% difference between week 1 and week 2 at point of harvest, (Fig. 4d). This difference will likely become negligible with additional repetitions. Overall good consistency was observed across the two weeks on the automated platform, proving the technical and biological robustness of the technique.

#### Application of the automated platform for screening studies

The aim behind the development of the platform was to speed up investigation of variables such as different strains, different protein carriers and different process conditions. The next step in establishing the automated platform was to prove the effectiveness of the technique for the screening of various conditions simultaneously.

##### Impact of *E. coli* strain on glycoconjugate formation

**Fig. 5:**
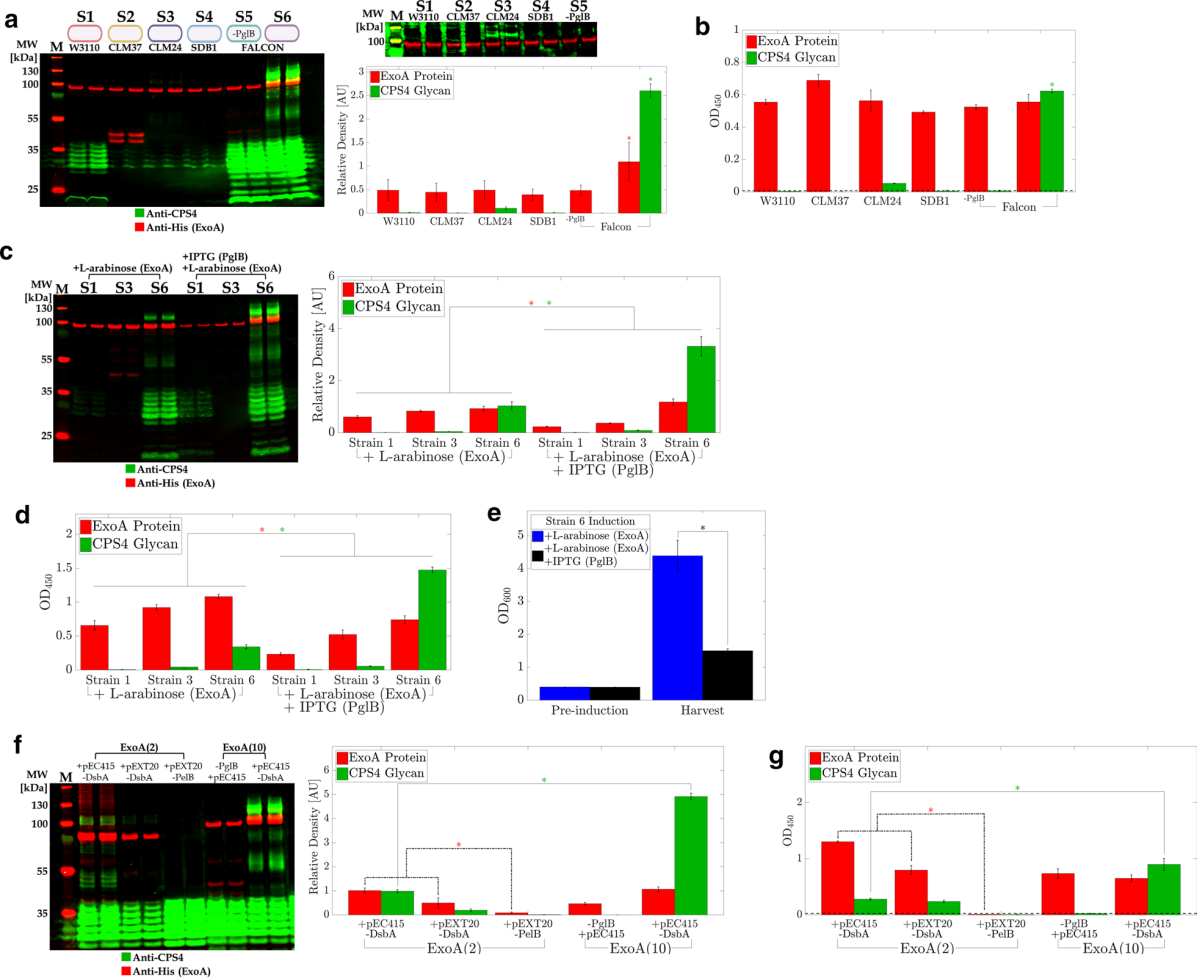
Automated screening comparing glycoconjugate expression across multiple strain variants cultured in parallel on the automated platform. **a**–**b** Comparison across five *E. coli* strains and a negative control that does not express PglB; **a** Standard and overexposed western blotting showing the presence of ExoA-CPS4 glycoproteins in periplasmic extracts and the subsequent densitometry analysis of the blots based on pixel intensity normalised to a control condition; **b** Sandwich ELISA analysis of periplasmic extracts shown in **a**. Dashed line showing background CPS4 glycan signal threshold from negative control strain without PglB; **c**–**e** Comparison across three strain variants with and without induction of PglB expression; **c** Western blotting and subsequent densitometry analysis; **d** Sandwich ELISA analysis of samples shown in **c**; **e** OD_600_ of Falcon (Strain 6) cultures, measured pre-induction and at harvest; **f**–**g** Comparison of Falcon strain expressing CPS4, PglB (or empty backbone for the negative control) and carrier protein ExoA with either 2 or 10 glycosylation sequons from either a pEC415 or pEXT20 plasmid backbone. Expression under a DsbA or PelB signal peptide was also compared; **f** Western blotting and subsequent densitometry analysis; **g** Sandwich ELISA analysis of extracts shown in **f**. Dashed line showing background CPS4 glycan signal threshold from negative control strain lacking PglB. Statistical significance (*) using an unequal variance two-tailed t-test is considered when *p* ≤ 0.05

Five different strains of *E. coli* were investigated in order to identify the most efficient at ExoA-CPS4 glycoconjugate production. In order of increasing number of mutations: W3110 (Strain 1); CLM37 (Strain 2); CLM24 (Strain 3); SDB1 (Strain 4); and Falcon (Strain 5 with no PglB ‘control’ or Strain 6), details of which are given in “Bacterial strains, growth conditions and plasmids” section and Table 1. All strains were transformed with three plasmid-encoded components essential for PGCT to work: (1) the glycan antigen; (2) the carrier protein; and (3) the PglB OST responsible for coupling glycans to protein substrates. The plasmids across the five strains were kept the same: pB4, responsible for the constitutive expression of the CPS4 glycan [31]; pEXT22-PglB, responsible for the IPTG inducible expression of the OST coupling enzyme PglB (or empty pEXT22 vector for the negative control, S5); and pEC415-ExoA(10), responsible for the l-arabinose inducible expression of the hyperglycosylatable version of carrier protein ExoA [27]. Each column of the 24-DSW plate was inoculated with 4 repeats of each strain and subsequently underwent all steps outlined in Fig. 3. Glycoconjugate expression was then analysed by SDS-PAGE/western blotting (Fig. 5a) and sandwich ELISA (Fig. 5b). The western blot analysis includes two replicates of each sample, annotated with Strain 1–6 (S1–S6) depictions. It is immediately apparent that Falcon (S6) shows higher levels of ExoA-coupled CPS4 glycan and ExoA carrier protein expression and exhibits a higher glycan/protein ratio. Densitometry analysis of ExoA-CPS4 expression based upon the pixel intensity normalised to a control condition confirms this. Between the protein and glycan components of the glycoconjugate produced, higher CPS4 glycan antigen content is desirable for the development and optimisation of the bioconjugate pneumococcal vaccine, thus the Falcon strain appears to be the most suited for vaccine development amongst the variants screened. Product expression in the remaining strains appears almost negligible (< 90% reduction of coupled CPS4 in comparison to the Falcon strain), however the strong intensity of the CPS4 signal with the Falcon strain saturates any apparent expression in the remaining strain variants, thus a separate western blotting excluding Falcon (S6) was made. It can now be seen that CLM24 (S3) has the second highest ExoA-coupled CPS4 yield with an approximate 94% decrease in the signal intensity to the Falcon strain. This result was confirmed by sandwich ELISA where CLM24 was shown to yield approximately 92% less ExoA-coupled glycan than Falcon, whilst the remaining strains do not exceed the ‘background’ threshold, denoted by the dashed line, provided by the no-PglB negative control (Strain 5, Fig. 5b). Increasing the culture volumes tenfold to 20 mL cultures showed all strains tested are indeed capable of producing ExoA-CPS4 glycoconjugates, however the genetic mutations of CLM24 and Falcon strains are clearly beneficial for production of the pneumococcal vaccine candidate (Additional file 2: Figure S2). Whilst the microscale cultures did not sufficiently identify the worst performers, the platform successfully screened for the best performing strains, thus rapidly pinpointing the conditions worth taking to scale-up experiments.

##### Impact of PglB expression on glycoconjugate formation

A second screening study was completed on the robot to investigate the effect of PglB induction on glycoconjugate formation. This is given in Fig. 5c–e. For this comparison the mother strain W3110 (S1) was selected against the two W3110-derivatives CLM24 (S3) and Falcon (S6). All samples were cultured in quadruplicate and induced with either l-arabinose alone to promote ExoA expression (Lanes 2–7 in the western blotting) or in combination with IPTG to also promote the expression of the PglB OST (Lanes 8–13). PglB in pEXT22 is under the control of an IPTG inducible *tac* promoter [32], so it is interesting to observe protein glycosylation even in the absence of IPTG. This is likely due to Lysogeny media containing traces of lactose, which lead to the mild induction of *tac* promoters, a phenomenon usually circumvented by using autoinduction (AI) or defined media formulations [33, 34]. This has enabled us to compare the effect of different PglB expression levels on glycoprotein formation. The glycoconjugate bands following induction using both IPTG and l-arabinose for the Falcon strain show a slight upward shift on the blot, indicating a heavier product from enhanced ExoA glycosylation and a larger glycan to protein ratio. Densitometry analysis of ExoA-CPS4 expression based upon the pixel intensity normalised to a control condition shows significantly higher glycosylation in the Falcon strain (S6, < 95% expression in S1 and S3) with a statistically significant boost in protein glycosylation following IPTG induction of PglB. This is again confirmed through analysis using a sandwich ELISA, where it is immediately apparent that PglB induction improves protein glycosylation and reduces protein expression for all the strains tested (Fig. 5d). Although glycoprotein formation and glycan coupling are boosted by inducing PglB expression, which is advantageous for a glycoconjugate vaccine, biomass yields at the point of harvest are reduced almost threefold for Falcon (Fig. 5e), similar to what was previously observed by Ihssen et al. [26] for different bioconjugates and strains. PglB induction caused a 10^4^ reduction in viable colony forming units per mL of culture (CFU/mL) in comparison to the uninduced sample, while IPTG induction on the negative control containing an empty pEXT22 vector did not affect biomass nor CFU/mL, indicating that higher expression of PglB exerts some toxicity to the cells (see Additional file 2: Figure S3). A decrease in the final biomass reduces the final volumetric productivity of the culture, however the features of the product obtained after PglB induction are more desirable for a glycoconjugate vaccine (higher glycan/protein ratio). Overall, it is evident that the Falcon strain exhibited significantly improved glycoconjugate expression over the other candidate strains investigated, while IPTG-mediated PglB induction was found to boost glycosylation of the carrier protein ExoA. This is considered a more desirable vaccine candidate as the amount of pneumococcal antigen per vaccine unit is increased. Further optimisation of the strain and plasmid components, as well as media composition, and concentration of inducers will be investigated to improve biomass yields.

##### Impact of ExoA constructs on glycoconjugate formation

Following the identification of the Falcon strain as the best ExoA-CPS4 glycoprotein producer, the impact of different ExoA constructs was investigated (Fig. 5f, g). A schematic representation of these constructs is provided in Additional File 2: Figure S4. Falcon were transformed with the same two plasmids pB4 and pEXT22-PglB, as previously described, responsible for the constitutive expression of the CPS4 glycan and the IPTG inducible expression of PglB, respectively. The third plasmid responsible for the expression of the carrier protein was either the l-arabinose inducible pEC415 or the IPTG inducible pEXT20. Both plasmids are medium copy vectors, however the plasmid copy number per cell of pEXT20 is higher (40 vs. 15–20 copies/cell). Additionally, the encoded ExoA was N-terminally fused to either DsbA or PelB signal peptides (where specifically mentioned, Sec pathway) to investigate their effects on periplasmic expression. Lastly, ExoA was modified to contain either 2 or 10 glycosylation sequons as a strategy to modulate the glycan content of the conjugates. This is denoted ExoA(2) or ExoA(10). From the western blotting analysis shown in Fig. 5f it is apparent that under the conditions tested the combination of plasmids where ExoA(2) is expressed from pEC415 leads to higher glycoprotein formation than when expressed from a pEXT20 backbone, and that almost no product is released into the periplasm from the PelB leader signal. The results confirm that increasing the number of glycosylation sites from 2 to 10 leads to enhanced ExoA glycosylation. Densitometry analysis of ExoA-CPS4 expression based upon the pixel intensity from the western blot shows a significant difference between the pEC415 and pEXT20 product expression levels and also between the DsbA and PelB periplasmic signals. An almost fivefold increase in glycosylation was shown between + pEC415-ExoA(2) and + pEC415-ExoA(10). Secondary analysis with ELISA showed similar results with DsbA clearly outperforming PelB as a periplasmic signal for ExoA (Fig. 5g). ExoA(10) was also shown to be threefold more glycosylated then ExoA(2), proving an effective strategy to adjust the amount of glycan antigen in the vaccine preparation. The pEC415 backbone shows a 3–5% increase in glycosylation in comparison to the pEXT20 construct, although a significant improvement is visible from the western blotting. Overall, this work showed the more favourable combination of the pEC415 backbone co-expressed with the DsbA signal for glycoprotein expression. Additionally, it showed that the amount of glycan antigen can be successfully modulated through the inclusion of a varying number of glycosylation sequons on the carrier protein.

##### Estimating the value and advantages of the automated platform

One of the greatest advantages of an automated platform, once proven fully effective in terms of successful and reproducible production, is the large reduction in manual handling hours for screening operations. With each screening experiment detailed in this work, a total of 6–8 different culture conditions were investigated, and 24 individual cultures were completed per experimental run. A conservative model Gantt chart of the manual working hours involved from the conception of preculture to the final western blot analysis is given in Additional file 2: Figure S5). This chart considers two scenarios; a manually handled culture from preculture to inoculation on a 24-DSW plate with the subsequent harvest, lysis and analysis steps over the course of one week, and the equivalent steps when an automated platform is employed. From this model a bar chart showing the amount of manual handling hours per well completed (see Additional file 2: Figure S6) shows that the use of an automated method results in approximately double the efficiency in comparison to manual handling. In addition, the robot is capable of unsupervised, walk-away screening studies, thus increasing researcher output within a given week. During the early research phase of vaccine development, this is a vital reduction in both time and monetary resources, speeding up the advancement and selection of vaccine candidates to preclinical and clinical trials.

## Conclusions

An automated microscale platform was developed to provide unsupervised ‘walk away’ operation, capable of screening multiple vaccine candidates in parallel. When developing higher throughput screening techniques, process robustness is paramount. Evaluation of the automated platform developed in this work showed overall good consistency both with technical and biological repeats. This proved successful integration with a numerical programming platform, which incorporated process adaptability and an automated decisional tool, in addition to liquid handling operations.

The desired application of the automated platform was the microscale screening of multiple conditions in parallel. Three separate screening studies are reported in this work. The first evaluated the differences in glycoconjugate expression and biomass for five different *E. coli* strains, whilst another investigated the impact of PglB expression on glycoconjugate formation. A clear improvement in product expression and glycosylation was found with the best producing Falcon strain upon induction of PglB. A third screening study investigated the impact of two different plasmid backbones, two periplasmic signals, and the number of glycosylation sites in ExoA on glycoprotein expression/recovery. The screening indicated that ExoA with a DsbA signal peptide expressed from a pEC415 l-arabinose inducible plasmid, in concert with PglB expressed from a pEXT22 backbone and pB4, leads to increased glycoprotein formation (at least in *E. coli* strain Falcon). Additionally, it was shown that the amount of CPS4 glycan antigen in the vaccine candidate increases with the number of glycosylation sites per carrier protein. Overall, 24 different culture conditions were investigated and a total of 120 individual cultures were completed. Minimal manual handling hours were needed to complete these experiments, highlighting the superiority and efficiency of higher throughput automated screening platforms, especially during early phase vaccine research.

The continuous feedback between the cell engineering aspect of this work and the design of the microscale platform is novel to the field of glycoengineering of vaccines. This collective approach speeds up the targeted development of operations most appropriate in advancing the early research phase of vaccines. The successful production of glycoconjugate vaccines in the biotechnology workhorse bacterium *E. coli* holds great potential in alleviating the cost burden currently associated with available vaccines targeted against pneumococcal disease. Understanding how to best optimise this system is paramount to develop a feasible bioconjugate alternative at a fraction of the current cost.

## Supplementary Information

**Additional file 1.** Additional tables.

**Additional file 2.** Additional figures.

## Data Availability

All data generated or analysed during this study are included in this published article [and its additional files].
