# Supplementary material for: Development of an automated platform for the optimal production of glycoconjugate vaccines expressed in Escherichia coli
Source: Microb Cell Fact. 2021 May 24;20:104. doi: 10.1186/s12934-021-01588-1 (PMC8142613; doi:10.1186/s12934-021-01588-1)
Supplement: Supplementary file 1 — Additional file 1. Additional tables. [file 12934_2021_1588_MOESM1_ESM.docx]

| **Strain** | **Genome** | **Source** |
| --- | --- | --- |
| *E. coli* W3110 | wbbL-, ƛ-, rph-1, IN(rrnD-rrnE)1 | [1,2] |
| *E. coli* CLM37 | W3110, ΔwecA | [3] |
| *E. coli* CLM24 | W3110, ΔwaaL | [4] |
| *E. coli* SDB1 | W3110, ΔwecA, ΔwaaL | [5] |
| *E. coli* Falcon | W3110, ΔlpxM ΔwecA-wzzE::galE | Kay *et al.* Manuscript in preparation |
| *E. coli* DH5α | F- Φ80lacZΔM15 Δ(lacZYA-argF) U169 recA1 endA1 hsdR17(rk-, mk+) phoA supE44 thi-1 gyrA96 relA1 λ- | New England Biolabs |

**Table S1:** Strains used in this study

| **Plasmids** | **Description** | **Source** |
| --- | --- | --- |
| pEC415 | Expression vector, L-arabinose inducible, Amp^R^ | [6] |
| pEXT20 | Expression vector, IPTG inducible (*tac* promoter), Amp^R^ | [7] |
| pEXT22 | Expression vector, IPTG inducible (*tac* promoter), Kan^R^ | [7] |
| pEC415-DsbA-ExoA(2) | Codon-optimized DsbA-ExoA(2) cloned into pEC415 | Recloned in this study, based on construct used in [8] |
| pEC415-DsbA-ExoA(10) | Codon-optimized DsbA-ExoA(10) cloned into pEC415 | Recloned in this study, based on construct used in [9] |
| pEXT20-DsbA-ExoA(2) | Codon-optimized DsbA-ExoA(2) cloned into pEXT20 | This study |
| pEXT20-PelB-ExoA(2) | Codon-optimized PelB-ExoA(2) cloned into pEXT20 | This study |
| pEXT22-PglB | *C. jejuni* PglB cloned into pEXT22 | Scott *et al.* Manuscript in preparation |
| pB4 | Recombinant expression of pneumococcal serotype 4 capsule, Tet^R^ | [10] |

**Table S2:** Plasmids used in this study

**References**

1. Liu D, Reeves PR. Escherichia coli K12 regains its O antigen. Microbiology. 1994;140:49–57.

2. Feldman MF, Marolda CL, Monteiro MA, Perry MB, Parodi AJ, Valvano MA. The activity of a putative polyisoprenol-linked sugar translocase (Wzx) involved in Escherichia coli O antigen assembly is independent of the chemical structure of the O repeat. J Biol Chem. 1999;274:35129–38.

3. Linton D, Dorrell N, Hitchen PG, Amber S, Karlyshev A V., Morris HR, et al. Functional analysis of the Campylobacter jejuni N-linked protein glycosylation pathway. Mol Microbiol. 2005;55:1695–703.

4. Feldman MF, Wacker M, Hernandez M, Hitchen PG, Marolda CL, Kowarik M, et al. Engineering N-linked protein glycosylation with diverse O antigen lipopolysaccharide structures in Escherichia coli. Proc Natl Acad Sci U S A. 2005;102:3016–21.

5. Garcia-Quintanilla F, Iwashkiw JA, Price NL, Stratilo C, Feldman MF. Production of a recombinant vaccine candidate against Burkholderia pseudomallei exploiting the bacterial N-glycosylation machinery. Front Microbiol. 2014;5:1–10.

6. Enggist E, Schneider MJ, Schulz H, Thöny-Meyer L. Biochemical and mutational characterization of the heme chaperone CcmE reveals a heme binding site. J Bacteriol. 2003;185:175–83.

7. Dykxhoorn DM, St. Pierre R, Linn T. A set of compatible tac promoter expression vectors. Gene. 1996;177:133–6.

8. Wacker M, Wang L, Kowarik M, Dowd M, Lipowsky G, Faridmoayer A, et al. Prevention of Staphylococcus aureus Infections by Glycoprotein Vaccines Synthesized in Escherichia coli. J Infect Dis. 2014;209:1551–61.

9. Marshall LE, Nelson M, Davies CH, Whelan AO, Jenner DC, Moule MG, et al. An O-Antigen glycoconjugate vaccine produced using protein glycan coupling technology is protective in an inhalational rat model of tularemia. J Immunol Res. Hindawi; 2018;2018.

10. Kay EJ, Yates LE, Terra VS, Cuccui J, Wren BW. Recombinant expression of Streptococcus pneumoniae capsular polysaccharides in Escherichia coli. Open Biol. 2016;6:150243.
