# Supplementary material for: Development of an automated platform for the optimal production of glycoconjugate vaccines expressed in Escherichia coli
Source: Microb Cell Fact. 2021 May 24;20:104. doi: 10.1186/s12934-021-01588-1 (PMC8142613; doi:10.1186/s12934-021-01588-1)
Supplement: Supplementary file 2 — Additional file 2. Additional figures. [file 12934_2021_1588_MOESM2_ESM.docx]

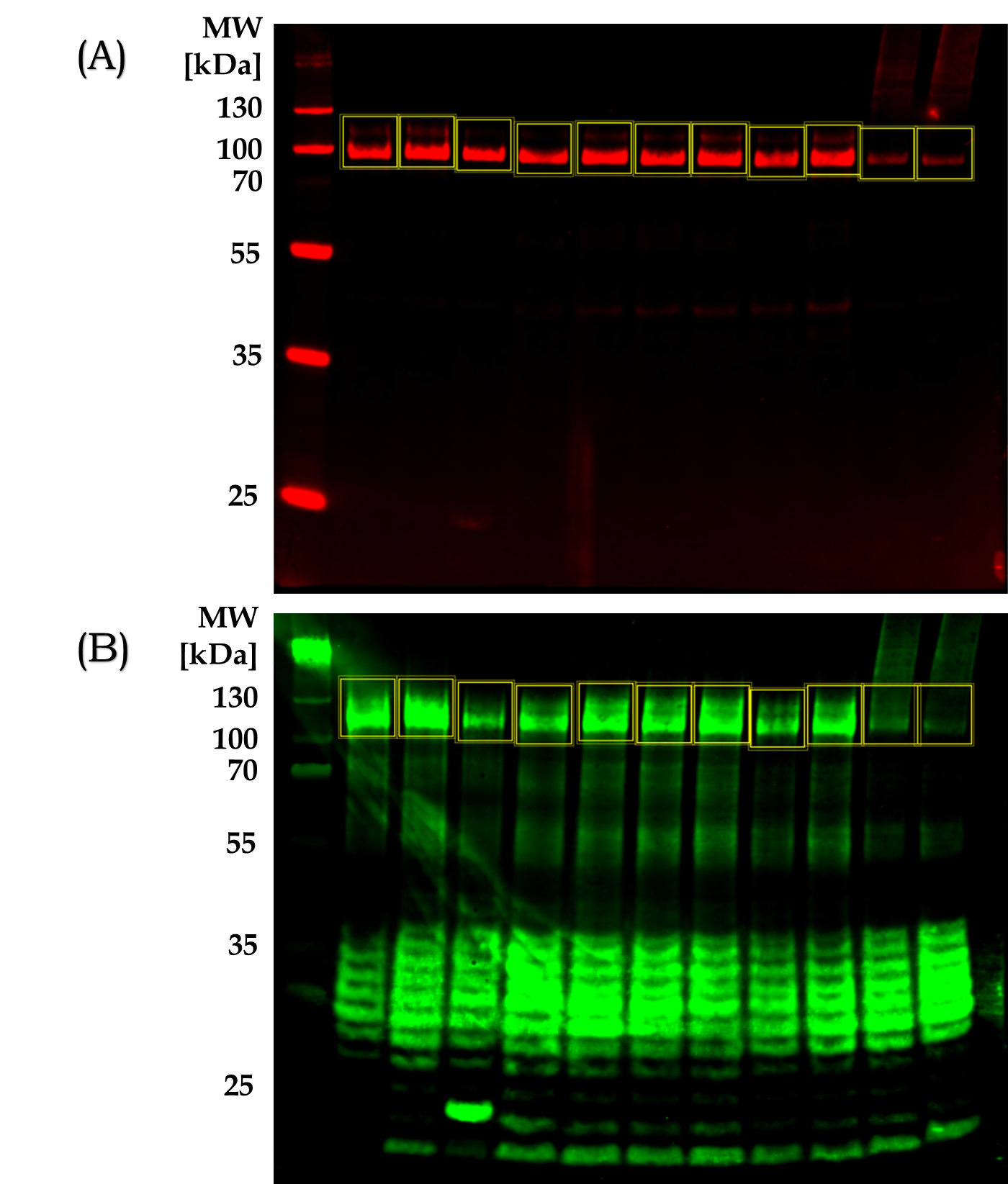


**Figure S1.** Exemplar semi-quantitative densitometry analysis using Image Studio Analysis tool of the ExoA-CPS4 glycoconjugate bands. **(A)** ExoA protein-component analysis in the 700 nm channel; **(B)** ExoA-coupled CPS4 glycan-component analysis in the 800 nm channel.


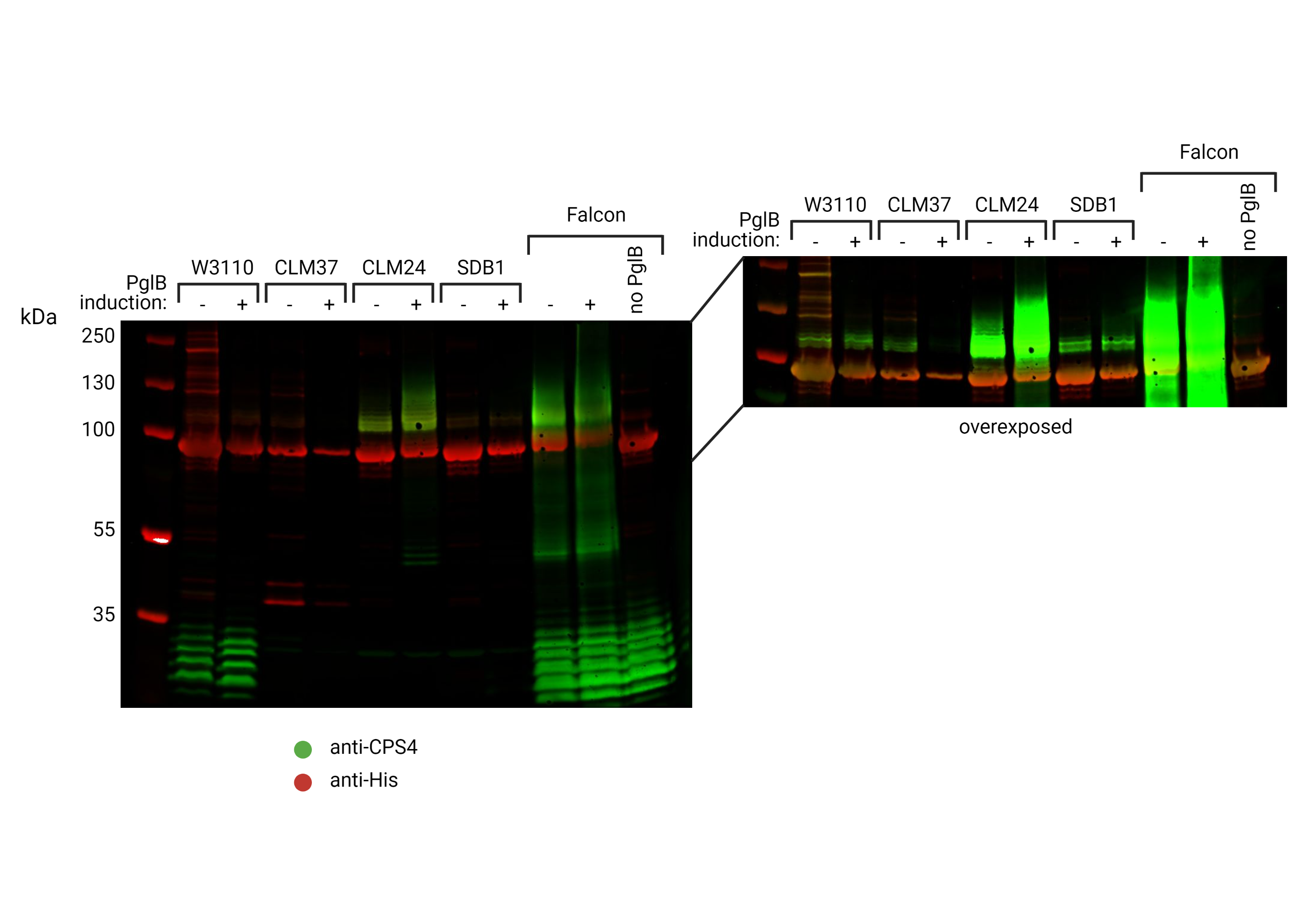


**Figure S2.** Western blotting showing glycosylation of ExoA(10) with CPS4 glycan in the five *E. coli* strains tested in this study. 2% (v/v) of periplasmic extracts obtained from 20 mL cultures were OD-matched and resolved on an SDS-PAGE, transferred to a nitrocellulose membrane and protein and glycan were stained with anti-his and anti-CPS4, respectively. ExoA(10) expression was achieved by L-arabinose induction across all samples, while PglB induction was promoted by IPTG induction in (+) samples. Enhancement of the 800 nm channel (right panel) shows that glycoconjugate formation is achieved in all the five strains tested, albeit protein expression and protein glycosylation efficiency differ clearly amongst strains and in absence (-) or presence (+) of PglB induction.


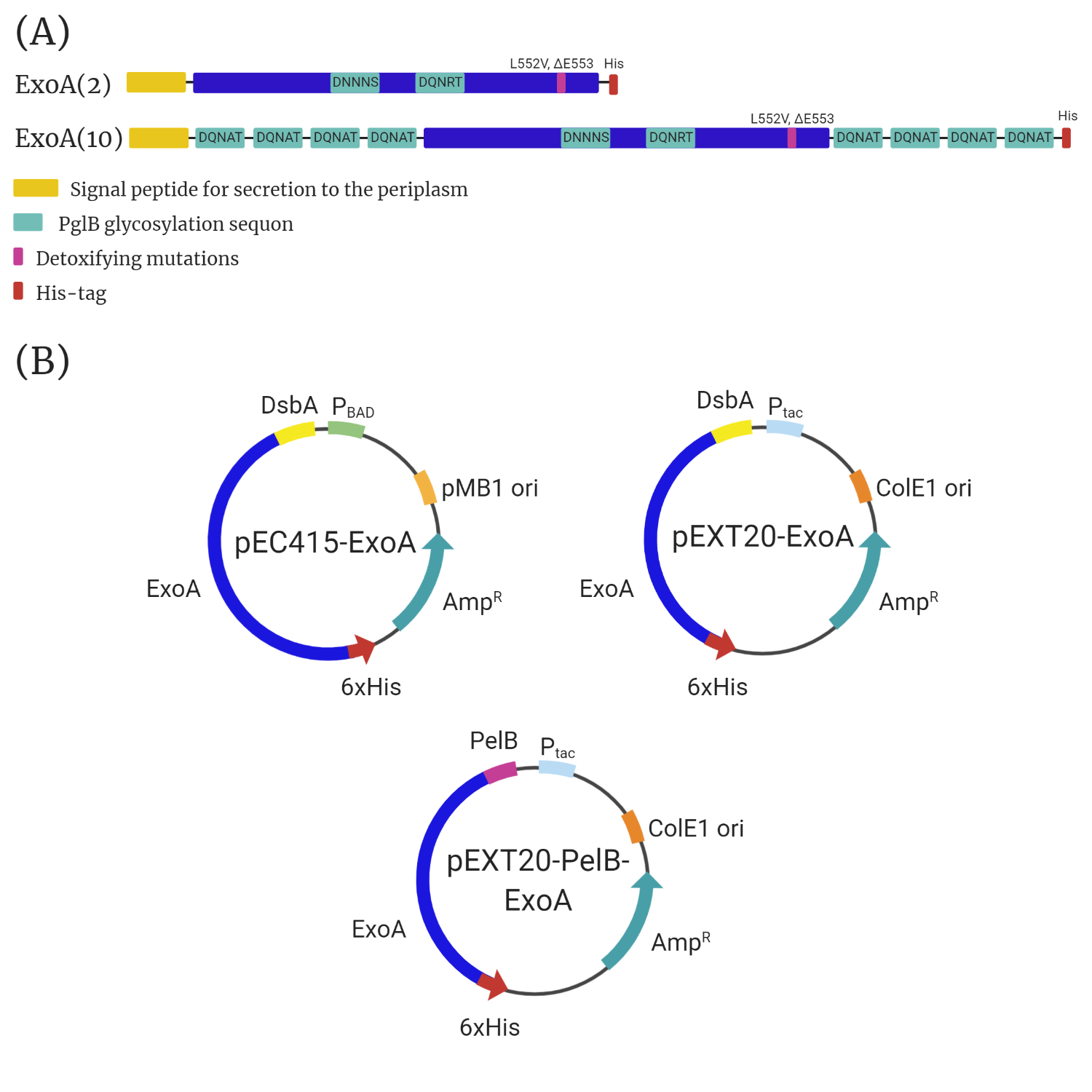


**Figure S3.** Carrier protein variants used in this study. **(A)** Schematic representation of ExoA protein variants; **(B)** Schematic representation of plasmids used, expressing either ExoA (2) or ExoA (10), under DsbA or PelB signal peptides for shuttling to the periplasm.


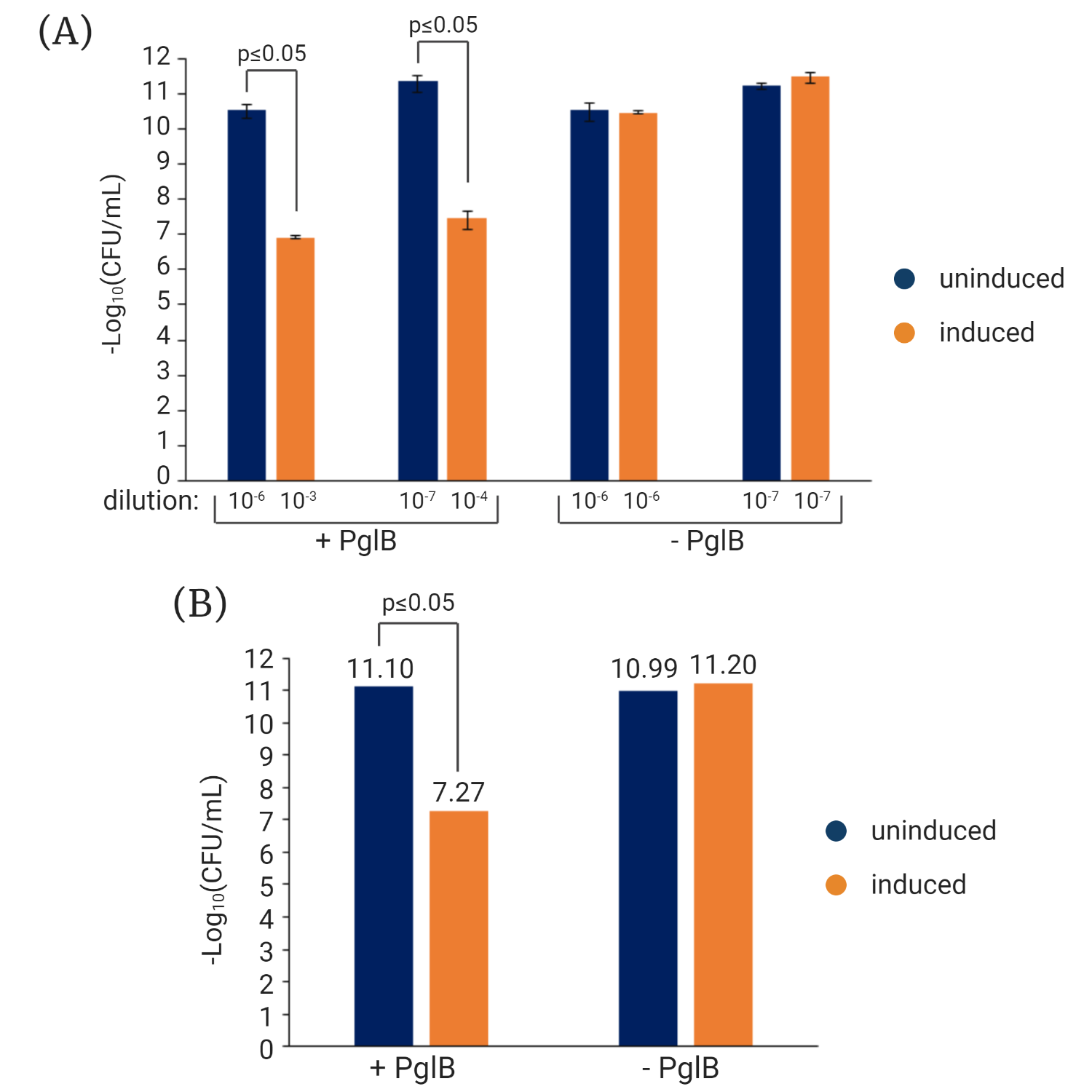


**Figure S4.** Induction of PglB causes a significant reduction in colony forming unit (CFU) counts. **(A)** Biological triplicates of Falcon transformed with pB4, pEXT22-PglB (or empty vector, -PglB control) and pEC415-ExoA (10) were induced with 0.4% (w/v) L-arabinose to promote expression of ExoA at OD_600_ = 0.6. Simultaneously, 1 mM IPTG was added to “induced” samples (orange) to promote the expression of PglB. No IPTG was added to “uninduced” samples (blue). CFU/mL were estimated for each sample at two dilutions at which colonies could be clearly counted. Averages and standard deviations (error bars) of biological and technical triplicates at each dilution are plotted. **(B)** Averages of CFU/mL calculated from the two dilutions plotted in **(A)** clearly indicating PglB induction causing a 3 – 4 -fold reduction in biomass, corresponding to ~ 4 Log_10_ reduction in CFU/mL. Statistical significance was considered when *p*≤0.05 using an unequal variance two-tailed t-test.


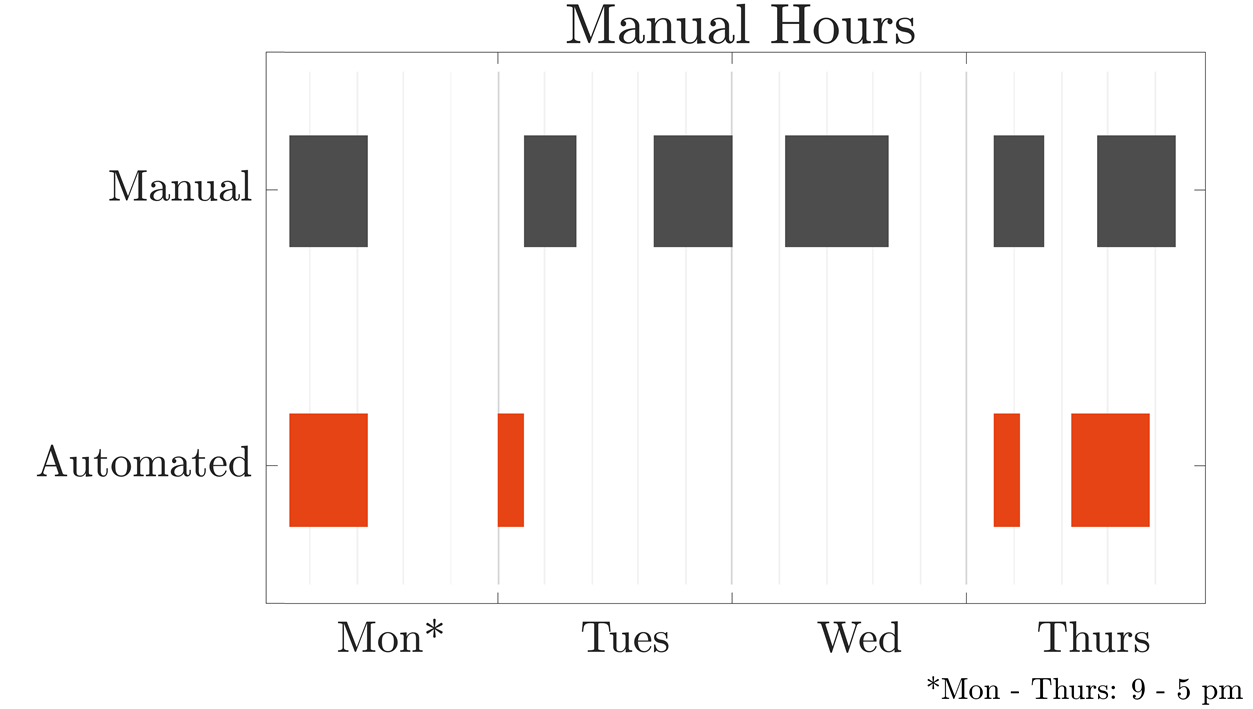


**Figure S5.** Comparison of manual handling time spent between fully automated and manual cultures; approximate Gantt chart showing manual handling hours from preculture (Monday) to western blot analysis (Thursday).


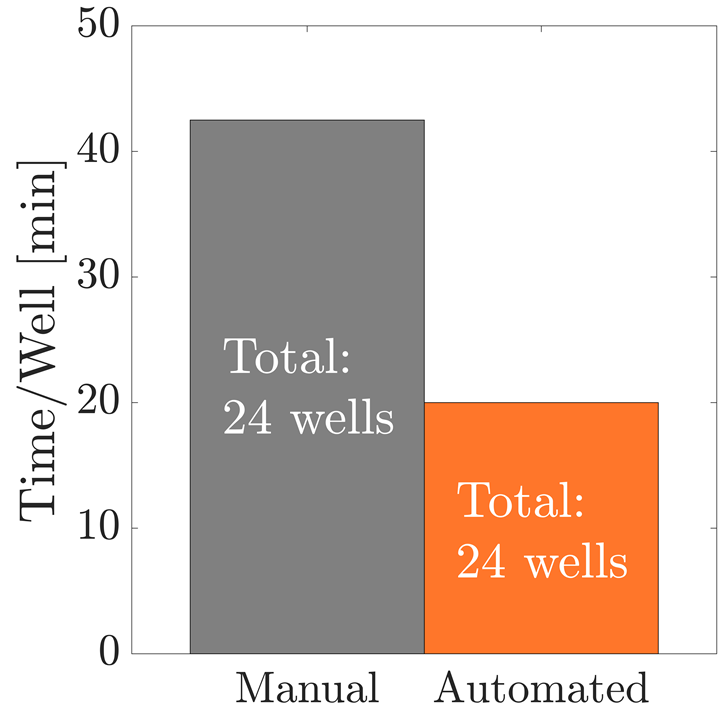


**Figure S6.** Comparison of manual handling time spent between fully automated and manual cultures; considered as manual handling time spent (minutes) per well per experimental run.
